# Acute upregulation of hedgehog signaling in mice causes differential effects on cranial morphology

**DOI:** 10.1242/dmm.017889

**Published:** 2014-12-24

**Authors:** Nandini Singh, Tara Dutka, Benjamin M. Devenney, Kazuhiko Kawasaki, Roger H. Reeves, Joan T. Richtsmeier

**Affiliations:** 1 Department of Anthropology, Pennsylvania State University, University Park, PA 16802, USA; 2 Johns Hopkins University School of Medicine, Institute of Genetic Medicine, Baltimore, MD 21287, USA; 3 Johns Hopkins University School of Medicine, Department of Physiology, Baltimore, MD 21205, USA

**Keywords:** Hedgehog signaling, Craniofacial shape, Down syndrome, Geometric morphometrics

## Abstract

Hedgehog (HH) signaling, and particularly signaling by sonic hedgehog (SHH), is implicated in several essential activities during morphogenesis, and its misexpression causes a number of developmental disorders in humans. In particular, a reduced mitogenic response of cerebellar granule cell precursors to SHH signaling in a mouse model for Down syndrome (DS), Ts65Dn, is substantially responsible for reduced cerebellar size. A single treatment of newborn trisomic mice with an agonist of the SHH pathway (SAG) normalizes cerebellar morphology and restores some cognitive deficits, suggesting a possible therapeutic application of SAG for treating the cognitive impairments of DS. Although the beneficial effects on the cerebellum are compelling, inappropriate activation of the HH pathway causes anomalies elsewhere in the head, particularly in the formation and patterning of the craniofacial skeleton. To determine whether an acute treatment of SAG has an effect on craniofacial morphology, we quantitatively analyzed the cranial form of adult euploid and Ts65Dn mice that were injected with either SAG or vehicle at birth. We found significant deformation of adult craniofacial shape in some animals that had received SAG at birth. The most pronounced differences between the treated and untreated mice were in the midline structures of the facial skeleton. The SAG-driven craniofacial dysmorphogenesis was dose-dependent and possibly incompletely penetrant at lower concentrations. Our findings illustrate that activation of HH signaling, even with an acute postnatal stimulation, can lead to localized dysmorphology of the skull by generating modular shape changes in the facial skeleton. These observations have important implications for translating HH-agonist-based treatments for DS.

## INTRODUCTION

The evolutionarily conserved Hedgehog (HH) signaling pathway and its ligands, desert hedgehog (DHH), Indian hedgehog (IHH) and Sonic hedgehog (SHH), are crucial for the development of a number of organs and cell groups (Cordero et al., 2006; Ingham and Placzek, 2006). In the craniofacial complex, the SHH pathway is implicated in the regulation of epithelial-mesenchymal interactions in the frontonasal process (FNP), and the anterior outgrowth and mediolateral expansion of the middle and upper jaw in prenatal development (Hu et al., 2003; Jeong et al., 2004; Marcucio et al., 2005; Hu and Marcucio, 2009a). Experimental studies using chick and mouse embryos have shown that disrupting SHH signaling in the forebrain alters the outgrowth of the facial prominences (Marcucio et al., 2005; Hu and Marcucio, 2009a; Hu and Marcucio, 2009b). A specific molecular boundary in the frontonasal ectoderm formed by SHH and fibroblast growth factor 8 (FGF8), called the frontonasal ectodermal zone (FEZ), controls expansion and outgrowth of the frontonasal prominence (Hu et al., 2003; Young et al., 2010). Early embryonic activation of SHH signaling in the forebrain results in a laterally expanded mid- and upper-face; conversely, a reduction in SHH signaling causes an elongated and narrow mid- and upper-face (Young et al., 2010). Such disruptions in embryonic SHH signaling in the forebrain and face mimic congenital craniofacial disease phenotypes – particularly midfacial dysmorphologies – in humans and mice (Chiang et al., 1996; Hu and Helms, 1999).

The majority of studies on the role of HH in craniofacial morphogenesis and disease have focused on the pathway during embryonic stages of development. In the present study, we examine the effects of a SHH pathway agonist, SAG 1.1 (SAG), on postnatal cranial development (Chen et al., 2002; Chen et al., 2004). SAG upregulates the HH pathway directly by binding to and activating the pathway regulator, Smo (downstream of the HH pathway receptor, Ptch) (Heine et al., 2011). Activation of the HH pathway via a single injection of SAG administered at birth rescued the morphology of the cerebellum and partially restored hippocampal functions in a mouse model of Down syndrome (DS), Ts65Dn (Davisson et al., 1993; Patterson and Costa, 2005). DS is a developmental disorder that occurs once every 700 live births, and is a result of an extra copy of human chromosome (Chr) 21 (Hsa21). One of the invariant and most debilitating phenotypic outcomes of DS is cognitive impairment, which is associated with reduced brain volume, particularly the size of the cerebellum (Reeves et al., 1995; Haydar and Reeves, 2012).

Mouse models of DS such as Ts65Dn have been used extensively to explore the genetic and developmental basis of phenotypes associated with DS (Reeves et al., 1995; Das and Reeves, 2011). Ts65Dn has an extra freely segregating marker chromosome that is the product of a reciprocal translocation between mouse chromosomes 16 and 17 (Mmu16 and Mmu17). This marker chromosome results in trisomy for over half of the human Chr 21 orthologs carried on Mmu16 and for the centromeric part of Mmu17, which includes 30–40 genes that are homologous to chromosomes other than Hsa21 (Davisson et al., 1993; Reinholdt et al., 2011). The Ts65Dn mice have abnormalities of the cerebellum, hippocampus and craniofacial skeleton that parallel the human DS phenotype (Baxter et al., 2000; Richtsmeier et al., 2000), all of which are replicated in the Dp(16)1Yey mouse, which is trisomic only for Hsa21 orthologs (Starbuck et al., 2014). Ts65Dn is the most widely studied mouse model for DS (Das and Reeves, 2011).

**TRANSLATIONAL IMPACT**

**Clinical issue**

The sonic hedgehog (SHH) pathway plays an important role during morphogenesis. Its dysfunction can cause a number of human developmental defects, such as cerebellar hypoplasia resulting from reduced response of granule cell precursors (GCPs) to the mitogenic effects of SHH, associated with Down syndrome (DS). Previous studies showed that a single treatment with an SHH pathway agonist (SAG), on the day of birth, rescued this defect and restored some cognitive functions in trisomic mice, a model of DS. In humans, treatment of newborns with high doses of glucocorticoids causes an analogous inhibition of GCP proliferation, and this can also be rescued in mice by using SAG. The translational potential of enhancing SHH signaling is intriguing. However, activation of this central signaling pathway that is involved in many aspects of development might have additional temporally specific and undesirable effects on other regions where SHH is expressed.

**Results**

In this study, the authors investigate the effects of SAG on craniofacial skeletal development in euploid (control) and trisomic mice. The results show that acute upregulation of the SHH pathway by a single treatment of SAG at birth causes significant deformation of adult craniofacial shape in euploid mice. The SAG-driven craniofacial dysmorphogenesis was dose-dependent and incompletely penetrant at lower concentrations. The observed deformations were most severe in the midline aspects of the facial skeleton compared with other parts of the cranium.

**Implications and future directions**

These observations have important clinical implications for translating SHH-agonist-based treatments for DS and other human conditions that involve the hedgehog pathway, and for understanding phenotypic variation driven by SHH signaling. The dose response on the craniofacial skeleton at postnatal day 1 suggests that lower doses of SAG might avoid effects on the skull, while still ameliorating cerebellar hypoplasia and producing the desirable improvements in cognitive function seen in

Cerebellar hypoplasia in Ts65Dn mice results from the reduced response of cerebellar granule cell precursors (GCPs) to the mitogenic effects of SHH, resulting in decreased numbers of granule cell (GC) and Purkinje cell neurons in the adult cerebellum (Dahmane and Ruiz-i-Altaba, 1999; Roper et al., 2006; Currier et al., 2012). Temporary upregulation of the HH pathway by a single injection of 20 μg/g SAG (μg SAG/g body weight) on the day of birth (P0) normalized cerebellar morphology in trisomic mice by postnatal day 6 (P6) (Roper et al., 2006). This amelioration lasted into adulthood, and was associated with restoration of some cognitive deficits characteristic of the mice (Das et al., 2013). In addition to the normalization of cerebellar morphology and the unexpected benefits to hippocampal-based learning and memory tasks in trisomic mice, SAG has also been shown to reverse cerebellar GC deficits caused by perinatal administration of glucocorticoids (Heine et al., 2011). Thus, SAG seems to have significant potential as a therapeutic molecule, especially for neural deficits. However, a subset of euploid mice that were treated with SAG showed obvious facial anomalies, reflecting the previously described crucial role of HH signaling in growth and development of the craniofacial complex (Hu and Helms, 1999; Tapadia et al., 2005; Cordero et al., 2006; Pan et al., 2013; Young et al., 2014) and indicating that activation of the HH pathway at the level of Smo by a pharmacological dose of SAG postnatally can result in craniofacial defects.

Here, we conduct an in-depth examination of the potential effects of the SHH pathway agonist SAG on postnatal craniofacial morphology, because the HH pathway plays a central role in a number of aspects of cranial growth and development. We use a quantitative approach to assess craniofacial shape changes in adult euploid and Ts65Dn mice that were injected with either 20 or 40 μg/g SAG or vehicle (Veh) on the day of birth (P0) to answer the following questions: (1) How is the shape of the adult facial skeleton, cranial vault and basicranium affected by acute postnatal stimulation of the HH pathway at birth? (2) How does dosage influence the effects of the agonist on craniofacial morphology? (3) How do these results inform translational research regarding the administration of SAG for DS-related phenotypes?

## RESULTS

### Canonical HH pathway expression shortly after birth

**Fig. 1.**
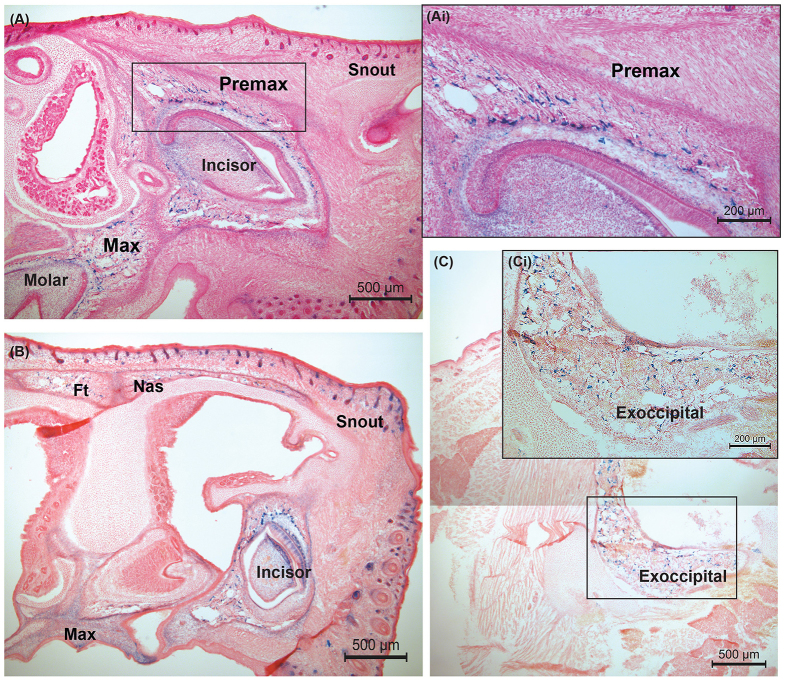
**Hedgehog signaling activity shown by *lacZ* expression in a P0 *Ptch1****^tm1Mps^***/J mouse, sectioned along the sagittal plane.** (A) Premaxilla (Premax), maxilla (Max) and teeth (incisor and molar). (Ai) Expression within the premaxilla shown at higher magnification. (B) Frontal (Ft) and Nasal (Nas) bones, incisor and maxilla. (C) Posterior cranium showing expression in the exoccipital region. (Ci) Exoccipital shown at higher magnification.

To first identify expression patterns of canonical HH during the hours after birth (P0), when the SAG injection is administered, we identified the regions expressing the receptor for HH family molecules and canonical pathway suppressor, Ptch, using P0 B6C3H *Ptch1^tm1Mps^*/J mice. These mice have a bacterial β-galactosidase gene (*lacZ*) ‘knocked in’ to the *Ptch1* locus, so that staining with X-gal identifies cells expressing the HH receptor at P0. We observed maximum expression of *Ptch1* in the facial skeleton, particularly around the premaxillae, maxillae and superior aspects of the nasal bones at P0, and some expression in the posterior basicranium in the exoccipital region (Fig. 1). The expression of *Ptch1* in these regions illustrates the areas where HH signaling is most active at P0, the age at which the SAG injection is administered. Other than the predominant activity of HH in the facial bones and minimal expression in the exoccipital bone (Fig. 1C), there is little or no other expression of *Ptch1* in other bones of the skull at P0 (Fig. 1).

### Morphometric analysis of SAG-injected and vehicle-injected mouse crania

We used three-dimensional (3D) coordinates of 41 landmarks to measure the craniofacial form (supplementary material Fig. S1a,b; Table S1) of a total of 56 adult wild-type (euploid) and trisomic mice (*n*=45 and 11, respectively) that had been injected with 20 μg/g SAG or Veh (Table 1; supplementary material Table S2) at birth. To extract cranial shape information, the landmarks were superimposed using generalized Procrustes analysis (GPA) (Dryden and Mardia, 1998). To analyze shape variation, we performed a principal component analysis (PCA) on the Procrustes shape coordinates (Slice, 2005). We also used a coordinate system-free method, Euclidean distance matrix analysis (Lele and Richtsmeier, 2001), to examine the localized variation in the crania of these mice (see Materials and Methods).

**Table 1.**
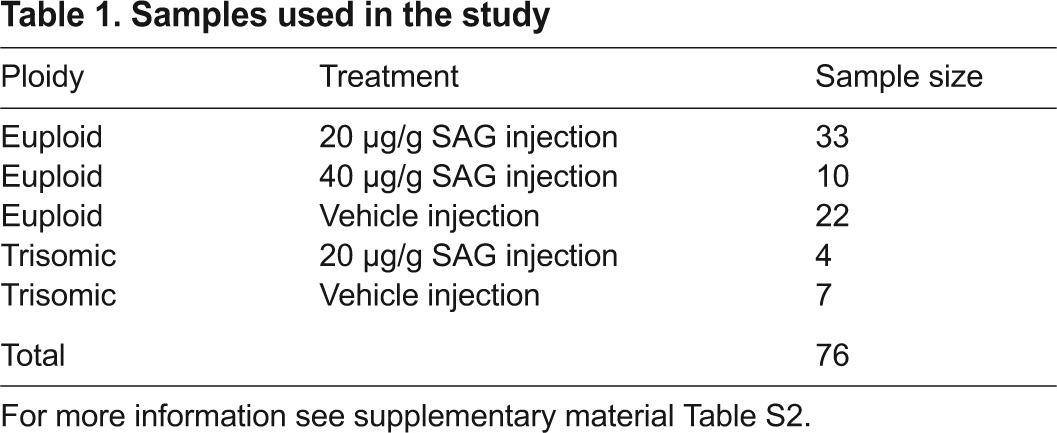
Samples used in the study

**Fig. 2.**
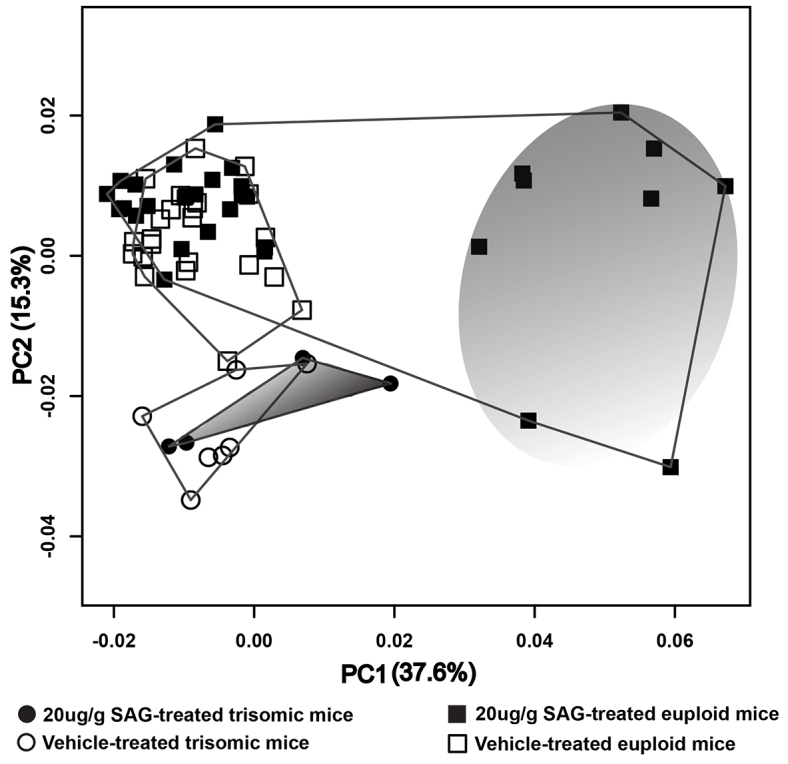
**Results of PCA based on Procrustes coordinates of 41 cranial landmarks viewed as a scatter plot of individual scores along PC1 and PC2.** The convex hulls represent the distribution of groups along the first two principal coordinate axes, PC1 and PC2. The majority of SAG-treated euploid mice cluster with the Veh-treated mice. A subset of euploid SAG-treated animals separate from the others along PC1, marked by the large gray shaded area. The small gray area indicates the distribution of SAG-treated trisomic mice. PC2 primarily separates all the trisomic mice from the euploid mice. PC2 also captures the within-group variation in the subset of euploid SAG-treated mice that separate along PC1.

**Fig. 3.**
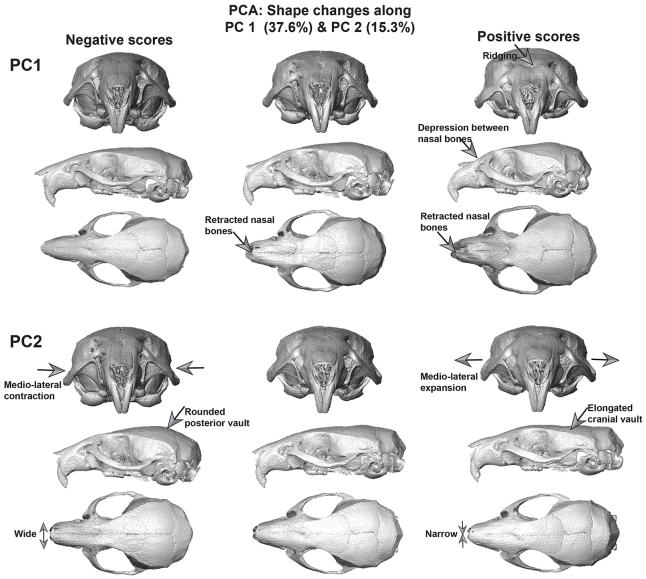
**Surface morphs representing shape variation along PC1 and PC2 as estimated by PCA of Procrustes coordinates of all 41 cranial landmarks.** The columns of surface morphs represent shape changes associated with negative and positive scores along the respective PC axes in Fig. 2. Views depicted for each axis are, from top to bottom: rostral, lateral and dorsal cranial views. The morphs on the negative end of PC1 represent the majority of the euploid and Veh-treated mice. The morphs in the middle column represent the approximate position (0.02) of the SAG-treated trisomic mice on PC1 (Fig. 2), and show slightly depressed and retracted nasal bones. The morphs on the positive end of PC1 represent the subset of 20 μg/g SAG-treated euploid mice and show deep indentation between, and ridging on, the lateral aspects of the nasal bones and overall retraction of the snout. Scores along PC2 illustrate shape changes between the trisomic (negative scores) and euploid (positive scores) mice. The morphs in the middle column represent specimens near −0.01 on PC2. The main shape variation along PC2 occurs along the supero-posterior aspect of the cranial vault, which is raised in the trisomic mice on the negative end of PC2 and comparatively flattened in the majority of the euploid mice occupying the positive end of the axis.

PCA results show a clear separation of nine of the 20 μg/g SAG-treated euploid mice from the remaining SAG- and Veh-injected euploid and trisomic mice along principal component 1 (PC1), accounting for 37.6% of the total variance in the sample (Fig. 2). The remaining SAG-treated euploid mice cluster with the Vehtreated euploid group, showing only minimal or no phenotypic effects of the agonist as characterized along PC1. Out of the four trisomic mice that were injected with 20 μg/g SAG, only one specimen plots close to the subset of SAG-treated euploid mice that show a morphological effect of the agonist on the positive end of PC1 (Fig. 2). The nine euploid mice treated with 20 μg/g SAG on the positive end of PC1 have retracted and deeply depressed nasal bones, wider snouts, some ridging along the lateral aspects of the fronto-nasal-premaxillary junction and a subtly raised posterior parietal region (Fig. 3). The single trisomic mouse associated with more positive scores along PC1 shares some of these features. PC2 (accounting for 15.3% of the total variance in the sample) mainly captures the distinctions between the euploid and trisomic mice, whether treated or not treated with SAG (Fig. 2), as well as variation among the nine affected SAG-treated euploid mice. The main shape differences between the euploid and trisomic craniofacial phenotypes captured by PC2 relate to changes in the neurocranium, which is rounded with a raised posterior cranial vault in the trisomic mice (and in two of the 20 μg/g SAG-treated euploid mice) (Fig. 3). PC2 also captures changes in the anterior aspect of the snout, which is slightly wider in the animals on the negative end of the axis, compared with the narrower snouts of the individuals on the positive end (Figs 2, 3). Similar differences between the skulls of Ts65Dn and euploid mice have been detailed in previous analyses (Richtsmeier et al., 2000).

**Fig. 4.**
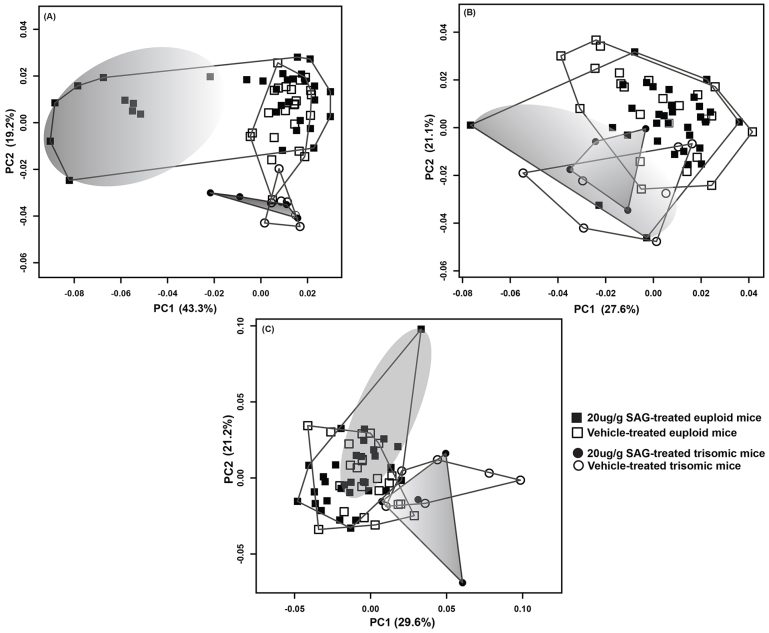
**Results of PCA based on Procrustes coordinates of different cranial components.** (A) PCA of 24 facial landmarks show a clear separation between a subset of SAG-treated euploid (large shaded gray area) and the remaining SAG and Veh euploid mice. The SAG-treated trisomic mice also vary along PC1, extending towards the SAG-treated euploid mice that show morphological effects of the agonist (small shaded gray convex hull). PC2 separates the euploid and trisomic groups and captures the within-group variation in the Veh-treated SAG and trisomic mice. (B) PCA plot of 7 cranial vault landmarks shows less separation among all the groups. PC1 accounts for the within-group variation among the groups, and PC2 illustrates the subtle differences between the SAG-treated euploid mice that do not show an effect of the agonist and Veh-treated mice on the positive end of the axis, and some of the euploid mice that show morphological effects of the agonist (shaded gray region) and the trisomic mice on the negative end (delimited by the smaller gray convex hull). (C) PCA plot of 10 basicranial landmarks. PC1 captures the differences between the euploid and trisomic mice, and PC2 captures the difference and within-group variation among the SAG-treated euploid (positive scores, marked by the shaded gray area) and trisomic mice (negative scores, marked by the shaded gray convex hull), primarily driven by one individual from each respective group.

To better identify localized shape changes, we subdivided the cranial landmarks into regions representing the facial skeleton, cranial vault and basicranium (supplementary material Fig. S1a; Table S1) and performed GPA and PCA on each cranial subunit separately (Fig. 4).

#### Facial skeleton

The subset of 20 μg/g SAG-treated euploid mice that were distinct in the previous analysis of all cranial landmarks, are also distinct when the facial landmarks are analyzed separately (Fig. 4A). PC1 (accounting for 43.3% of the total variance in the sample) separates the subset of nine dysmorphic SAG-treated euploid mice from the remaining SAG- and Veh-treated animals. These nine euploid SAG-treated mice (and to an extent, two trisomic mice) have a relatively retracted anterior snout (supplementary material Fig. S2). PC2 (19.2%) captures the differences between the euploid and trisomic facial morphology (Fig. 4A), and is associated with changes in the lateral aspects of the face (supplementary material Fig. S2).

#### Cranial vault

A separate PCA of the cranial vault landmarks does not distinguish among the groups (Fig. 4B). The range of variation on the negative end of PC1 (27.6%) is driven by one affected SAG-treated euploid specimen and a Veh-treated trisomic mouse, and the positive end is occupied primarily by the unaffected and Veh-treated euploid mice (Fig. 4B; supplementary material Fig. S3). Craniofacial morphology of the SAG-treated trisomic mice overlap with the Veh-treated trisomic mice and the euploid groups, showing no overt effects of the agonist in the cranial vault. PC2 (21.1%) shows overlap among all the groups, but the majority of the unaffected and Veh-treated euploid mice occupy the positive end of the axis.

#### Basicranium

PC1 (29.6% of the total variance) separates the trisomic from the euploid mice (Fig. 4C; supplementary material Fig. S4). PC2 (21.2%) captures the differences between the SAG-treated euploid mice and SAG-treated trisomic mice, but this is primarily driven by one individual from each group occupying the positive and negative ends of the axis, respectively (Fig. 4C). It is worth noting that, unlike in the PCA of the facial landmarks, only three out of nine SAG-treated euploid mice that were distinct in the analysis of the entire skull were distinct in the analysis of the cranial vault and basicranium landmarks.

### Frequency and magnitude of craniofacial effects is dose-dependent

**Fig. 5.**
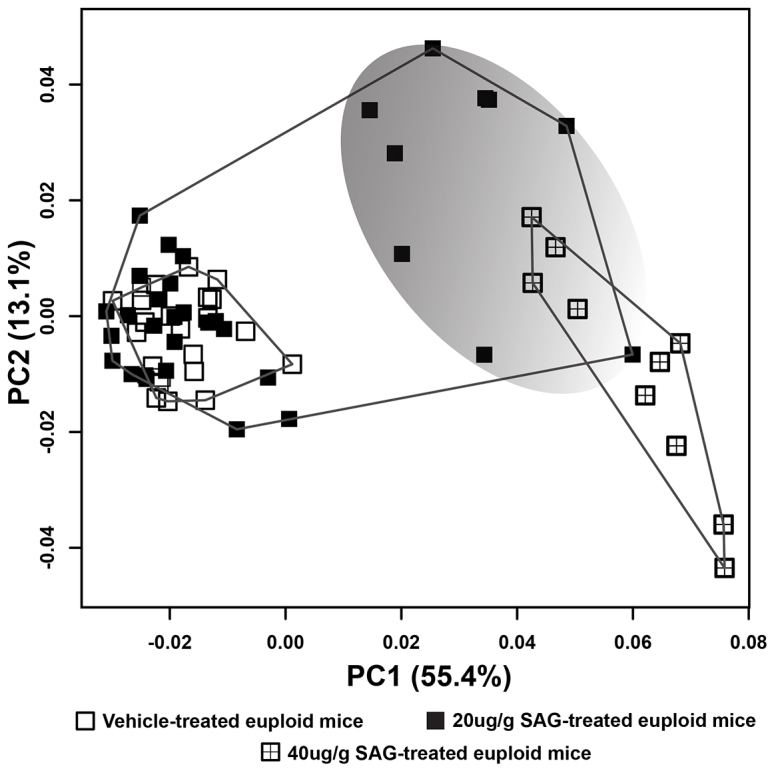
**Results of PCA based on Procrustes coordinates of 41 cranial landmarks.** Scatter plot of individuals according to their scores along the principal component axes reveal that PC1 separates all the 40 μg/g SAG-treated and a subset of the 20 μg/g SAG-treated euploid mice (marked by the shaded gray area) from the remaining SAG- and Veh-treated euploid animals. PC2 mainly captures the within-group variation among the SAG-treated mice, distinguishing between the lower (marked by the shaded gray area) and higher dosed specimens.

We hypothesized that the absence of morphological effect in 72% of the SAG-treated euploid animals was due to a dose-dependent threshold effect. To further examine this hypothesis, we injected an additional ten mice with 40 μg/g SAG and ten with Veh during the same experiment. These mice were imaged and the data were added to our analyses. Results of PCA (Fig. 5) based on the Procrustes coordinates of the 41 cranial landmarks show that euploid mice treated with 40 μg/g SAG and the subset of nine euploid mice that showed morphological changes after treatment with 20 μg/g SAG group together along PC1 (55.4%) and separate from the remaining mice in the sample. PC2 (13.1%) mainly captures the variation among the subset of nine mice treated with 20 μg/g SAG that show morphological effects of the agonist and the 40 μg/g SAG dosed mice, with individuals that were given the higher dose of SAG occupying the positive extreme of PC1 and the negative extreme of PC2. Shape variation of the entire cranium characterized by PC1 and PC2 is similar to that seen in the analysis of the 20 μg/g SAG dosed animals (Fig. 3), but the neurocranial shape changes are more exaggerated in the higher-dosed specimens. A PCA analysis that includes the trisomic mice and all euploid mice (both 20 μg/g and 40 μg/g SAG-treated) is included in the supplementary materials (supplementary material Fig. S5).

In addition, to examine whether the dosage of SAG has any effect on postnatal size in the euploid and trisomic mice, we performed multivariate regression analyses (supplementary material Fig. S6) of the 41 Procrustes shape coordinates on centroid size to analyze size-related shape changes (i.e. allometric variation) in the groups. The biggest difference in size was between the euploid and trisomic mice, a finding already documented in Ts65Dn mice that parallels the effects of trisomy 21 in humans with DS. Within the euploid mice, the subset of nine 20 μg/g SAG-treated mice that showed a morphological effect of the agonist (supplementary material Fig. S6A) and the mice that were given the 40 μg/g dose of the agonist (supplementary material Fig. S6B) were slightly smaller in size than all the other SAG-treated euploid mice. However, we did not find any size-related shape differences between the SAG- and Veh-treated trisomic mice.

## DISCUSSION

Our quantitative analysis of the craniofacial skeleton examines the effects of a HH pathway agonist shown previously to ameliorate brain abnormalities in a mouse model of DS (Roper et al., 2006; Das et al., 2013). In addition to the positive effects of acute SAG treatment on DS-related cerebellar features and function, we now document an important additional effect of this postnatal perturbation of the HH pathway on overall cranial shape, especially of the facial skeleton. The effects on the craniofacial skeleton were not the focus of the original experiment that targeted the morphology and function of the cerebellum (Das et al., 2013). This is the first demonstration of a localized effect of HH signaling on postnatal development of the craniofacial skeleton initiated by a measured perturbation on the day of birth. An acute dose of SAG that is likely dispersed within a day of administration has a large effect on the adult facial morphology.

The β-galactosidase-stained sections of a *Ptch1^tm1Mps^* reporter mouse effectively identified cells responsive to the canonical HH pathway at P0, and these correlate with the regions most affected by the SAG agonist in euploid mice at P0. Expression of *Ptch1* was predominantly found in the nasal bones, premaxillae, maxillae and the anterior portion of the frontal bone, and to a lesser extent in the developing occipital bone, indicating the structures most responsive to upregulation of HH at P0. A previous study (Mak et al., 2008) using *Ptch^1c/−^;HOC Cre* mice to determine HH signaling activity in postnatal bone formation found strong *lacZ* expression in early differentiating osteoblasts and reduced expression in mature osteoblasts and osteocytes in the humerus (Mak et al., 2008). Although Mak et al. (Mak et al., 2008) focused on later postnatal ages (P5) and examined the postcranial skeleton that forms endochondrally, their results help to define the specific cell types that are most responsive to the HH pathway during postnatal bone development.

In accordance with the *lacZ* findings, morphometric analysis of the adult craniofacial skeleton showed increased shape variation in the snout, particularly in the midline structures of the facial skeleton in both the euploid and trisomic SAG-treated mice that showed an effect of the agonist. Among the mice that were given the lower dose of the agonist, only a subset exhibited changes in cranial shape, whereas all the mice administered the higher dose showed dysmorphology of the cranium. These results suggest a threshold effect of drug dosage on cranial dysmorphogenesis. It will be critical to determine the lowest dose capable of normalizing cerebellar morphology and improving hippocampal function while avoiding effects on the craniofacial skeleton. A further variable here is that the mice are maintained as an advanced intercross between B6 and C3H, so genetic variation among these individuals might contribute to the observed morphological variation. Assessment of additional genetic risk factors (individualized medicine) might ultimately indicate which individuals should or should not participate in this type of therapeutic regimen, or whether a dosage can be individually prescribed.

The morphological changes documented among the affected mice illustrate modular and targeted patterns of shape change that are specific to the facial skeleton. Our findings are consistent with previous quantitative studies that tested varying levels of *Shh* expression in chick embryos and found that an increase of SHH in the forebrain resulted in wider and shorter mid-faces, capturing phenotypic variation in the mediolateral growth axis of the FNP (Hu et al., 2003; Young et al., 2010). The expression domain of SHH in the developing neurocranium is ill-defined and debatable. Some studies suggest the presence of *SHH* gene expression in the osteogenic fronts of the midline (sagittal) suture mesenchyme (Kim et al., 1998), whereas others show little to no expression of *SHH* in the midline suture mesenchyme (Lenton et al., 2011) in mice. SHH expression has been recorded near the spheno-occipital synchondroses of developing mice (Pan et al., 2013). An overexpression of SHH can increase expression of the IHH inhibitor, PTHrP, affecting normal growth of the cranial base in the later stages of development (Karp et al., 2000; Tavella et al., 2004).

Because SAG activates the pathway downstream of Ptch, the specific ligand, whether IHH or SHH, is not crucial here. However, our findings suggest that the majority and most severe morphological changes after treatment by SAG occur in the face, and we attribute these changes mainly to disruptions in SHH signaling. Still, because SHH and IHH have overlapping expression domains and control different aspects of cranial base formation (Nie et al., 2005), our data do not allow us to eliminate a potential role for IHH in the effects of SAG on postnatal endochondral bone growth and patterning. IHH is closely related to SHH and promotes osteogenesis by stimulating osteoblast differentiation of mesenchymal cells (Shimoyama et al., 2007), and regulates chondrocyte differentiation and endochondral bone formation (Vortkamp et al., 1996; St-Jacques et al., 1999; Long et al., 2001; Cohen, 2003; Tavella et al., 2004; Young et al., 2006). Studies have also indicated the involvement of IHH in cranial vault bones that are primarily derived from intramembranous ossification (Jacob et al., 2007; Lenton et al., 2011; Pan et al., 2013), but not in the facial skeleton. Previous analysis of skulls of neonate *Ihh^−/−^* mice revealed cranial vault defects such as rounded neurocrania, small frontal and parietal bones and an expansion of the sagittal suture compared with their wild-type littermates (St-Jacques et al., 1999; Lenton et al., 2011); however, loss of IHH did not impact cell proliferation in the intramembranous bones (Lenton et al., 2011).

Not only does SAG affect craniofacial shape, it also impacts craniofacial size. The affected 20 μg/g and all the 40 μg/g SAG-treated euploid mice were smaller in overall cranial size compared with the unaffected and Veh-treated animals. Although our focus has been on allometric variation, i.e. size-related shape changes, our findings suggest that acute (temporary) upregulation of the HH pathway by SAG impacts postnatal growth by decreasing the overall size of the craniofacial structures. This conflicts with previous reports of a reduction specifically in SHH signaling causing decreased head size in chick embryos, particularly decreased growth in the facial mesenchyme (Ahlgren and Bronner-Fraser, 1999; Marcucio et al., 2005; Young et al., 2010). Because our focus here is on the effects of an acute, temporary upregulation of HH at P0 on the adult phenotype, our findings are not entirely comparable to results from embryonic data.

Interestingly, we did not find the same allometric pattern in the SAG-treated trisomic mice compared with the Veh-treated ones, suggesting that SAG did not affect size in our sample of SAG-treated trisomic mice. Both SAG- and Veh-treated trisomic mice were considerably smaller in size than the euploid mice, corroborating previous quantitative findings that have shown the Ts65Dn skull to be smaller than the skulls of euploid mice (Richtsmeier et al., 2000; Olson et al., 2004).

Our work identifies a caveat to be dealt with in translating SAG for therapeutic use as a strategy for ameliorating cognitive effects of DS. A single exposure to SAG at birth normalized the size of the cerebellum and rescued hippocampal functions in trisomic mice (Das et al., 2013). SAG is a small molecule that crosses the gut, placenta and blood-brain barrier and so can potentially activate the canonical HH pathway in any cell where Smo is expressed. Improvement in one aspect of the phenotype can be associated with additional, undesirable effects on other phenotypes. This is especially true when therapeutics are administered during early stages of active tissue development. Without a full understanding of the multitude of interactions that occur during development (e.g. genetic, cell-cell, tissue-tissue) it is difficult to anticipate the unintended targets of therapeutic agents. Beyond the role of SAG in therapeutic medicine, our finding of differential effects of HH pathway activation on three regions of the cranium provides insight into the varied response of cranial units to disruptions in signaling at birth and during postnatal development. A better understanding of the rules of development underlying the relationship between the modular response to systemic changes in signaling and associated phenotypic variation is required to inform clinical research on craniofacial disorders as well as investigations into the patterns of evolutionary developmental change of cranial phenotypes.

## MATERIALS AND METHODS

### *lacZ* staining protocol

For the β-galactosidase (*lacZ*) staining of P0 *Ptch1^tm1Mps^*/J mice, each mouse was collected within a few hours of birth. The head was removed, washed in 1× PBS, and drop fixed in 4% PFA overnight. The head was next washed twice in 1× phosphate buffered saline (PBS) and cryoprotected in 0.5 M sucrose for 4 days. The head was positioned for sagittal sectioning, frozen in OCT compound (Tissue-Tek^®^, Sakura Finetek, Torrance, CA, USA) in peel-away cryomolds using a dry ice and ethanol bath, and stored at −80°C. The head was sectioned with a cryostat in 10-μm sagittal sections. The sections were kept at 10-μm every 5th or 6th section. Cryosections were fixed for 15 minutes in 0.1 M phosphate buffer with 0.2% glutaraldehyde and 2% formaldehyde, washed twice for 5 minutes in 0.1 M phosphate buffer with 2 mM MgC1_2_, and stained at 37°C for 24 hours in X-gal mixer containing 2 mM MgCl_2_, 5 mM potassium ferrocyanide, and 5 mM potassium ferricyanide supplemented with 1 mg/ml X-gal (Thermo Fisher Scientific Fermentas, Pittsburgh, PA, USA). After staining with X-gal, slides were washed twice for 5 minutes in 1× PBS and counterstained for 30 minutes in nuclear fast red (Sigma-Aldrich, St Louis, MO, USA). Slides were then rinsed twice in 1× PBS. The tissue was finally successively dehydrated in ethanol, cleared with xylenes, and protected with coverslips attached using Permount (Thermo Fisher Scientific Fermentas, Pittsburgh, PA, USA).

### Sample

To visualize the expression of HH signaling activity shown by *lacZ* expression, B6;129-*Ptch1^tm1Mps^*/J mice were obtained from The Jackson Laboratory, backcrossed for five generations onto a B6 background, at which point they were crossed to C3H mice to create an F1 generation, B6C3H. *Ptch1^tm1Mps/+^* mice were maintained in the Reeves’ laboratory colony as a B6 × C3H advanced intercross and only one individual was used exclusively for illustrative purposes shown in Fig. 1.

For the PCA analyses of skull morphology, six cohorts of SAG- and Vehtreated B6×C3H advanced intercross euploid mice were utilized, making up a total sample of 76 animals (Table 1 and supplementary material Table S2), including the Ts65Dn mice. Alternate euploid litters received Veh or SAG at 20 or 40 μg/g injected subcutaneously at the back of the skull within hours of birth. Further detail on breeding and dosing of the 76 euploid and Ts65Dn animals used in morphometric analyses is provided in supplementary material Table S2. Between 10 and 18 weeks of age mice were anesthetized with isoflurane and euthanized by cervical dislocation or perfusion. Heads were removed and placed in 4% paraformaldehyde (PFA) for at least 48 hours. The skulls were then washed and stored in PBS at 4°C until they were scanned by micro-computed tomography (μCT) for morphological assessment. All procedures were reviewed, approved and carried out in compliance with animal welfare guidelines approved by the Johns Hopkins University and the Pennsylvania State University Animal Care and Use Committees.

### Imaging and morphometric analysis

μCT images were acquired for 76 adult mouse skulls at the Johns Hopkins Medical Institutions through the Small Animal Resource Imaging Program at the Research Building Imaging Center using a Gamma Medica X-SPECT/CT scanner (Northridge, CA, USA) with 0.05 mm resolution along *x*, *y* and *z* axes for each mouse (supplementary material Table S2). Image data were reduced from 16-bit to 8-bit for image analysis. Isosurfaces were reconstructed to visualize all cranial bones using the software package Avizo 6.3 (Visualization Sciences Group, VSG). Forty-one cranial landmarks (supplementary material Fig. S1) were located on the isosurfaces and their three-dimensional (3D) coordinate locations were used in morphometric analyses. To establish repeatability of landmarks and evaluate intra-observer measurement error, we re-measured five SAG-treated and five Veh-treated mice at the end of data collection. Measurement deviations between the two trials were limited to 0.03 mm.

The landmark configuration of all specimens were superimposed using generalized Procrustes analysis (GPA) that extracts Procrustes shape coordinates from the original landmark data by translating, scaling and rotating the data, subsequently yielding a measure for size called centroid size and a description of the shape variation in the sample without the effects of isometric size (Dryden and Mardia, 1998; Slice, 2005). In addition to analysis of the overall cranial shape using 41 cranial landmarks (supplementary material Fig. S1a; Table S1), subsets of landmarks representing the facial skeleton (24 landmarks), cranial vault (7 landmarks) and basicranium (10 landmarks) were analyzed separately (supplementary material Table S1). We additionally conducted a separate analysis (not shown) with just a subset of ten facial landmarks (supplementary material Table S1) to ensure that the larger number of landmarks in the face (i.e. 24) compared to the cranial vault and basicranium does not influence the result. For each of these analyses, the subset of landmarks was superimposed using GPA and the resulting Procrustes coordinates were submitted to principal component analysis (PCA). PCA of Procrustes coordinates is based on an eigenvalue decomposition of a covariance matrix, which transforms the Procrustes coordinates into scores along principal components (PCs) (Slice, 2005). The PCs are not correlated with one another and the first PC accounts for the maximum amount of variation in the sample, PC2 accounts for the next highest amount of variation, with each subsequent PC accounting for the next highest amount of variation. In most cases, the first few PCs explain most of the variance in the dataset. Craniofacial shape variation as analyzed by PCA was visualized on surface scans and via wireframe diagrams. The surface scans were generated in AVIZO 6 (Visualization Sciences Group, VSG) by warping the principal component scores onto the mean shape of the respective SAG- and Veh-treated groups, and the wireframes were constructed in MorphoJ (Klingenberg, 2011). To examine allometric variation in our dataset, we conducted multivariate regression analysis of Procrustes shape coordinates of all the cranial landmarks on centroid size. All analyses were conducted in the programming software R version 3.1.0 (The R FAQ; http://cran.r-project.org/doc/FAQ/R-FAQ.html) and MorphoJ.

In addition, we analyzed all landmark data using Euclidean distance matrix analysis (EDMA) (Lele and Richtsmeier, 2001). EDMA is a coordinate system-free morphometric method that enables statistical testing of the shape differences between groups, as well as a statement on the variability of form difference estimators contained within confidence intervals based on the model independent bootstrap method. EDMA converts 3D landmark data into a matrix of all possible linear distances between unique landmark pairs and tests for statistical significance of differences between shapes using non-parametric confidence intervals (Lele and Richtsmeier, 2001). We tested for morphological differences in each dosage group compared with the Veh-treated animals and we tested for differences in the 20 μg/g SAG to Veh comparison and the 40 μg/g SAG to Veh comparison. In general terms, EDMA results correspond with the Procrustes-based analyses reported here. Moreover, confidence intervals for the comparison of specific linear distances between groups strongly supported a dosage effect of the SHH agonist. Animals receiving SAG at 40 μg/g show differences of greater magnitude and also a larger number of linear distances that are significantly different from Veh-treated mice relative to the comparison of animals receiving SAG at 20 μg/g with Veh-treated mice.

## Supplementary Material

Supplementary Material
