# Supplementary material for: Acute upregulation of hedgehog signaling in mice causes differential effects on cranial morphology
Source: Dis Model Mech. 2014 Dec 24;8(3):271–9. doi: 10.1242/dmm.017889 (PMC4348564; doi:10.1242/dmm.017889)
Supplement: Supplementary Material [file supp_8_3_271__index.html]

Acute upregulation of hedgehog signaling in mice causes differential effects on cranial morphology — Supplementary Material 

# Acute upregulation of hedgehog signaling in mice causes differential effects on cranial morphology

## DMM017889 Supplementary Material

**Files in this Data Supplement:**

- **Supplementary Material**
