# Supplementary material for: Acute upregulation of hedgehog signaling in mice causes differential effects on cranial morphology
Source: Dis Model Mech. 2014 Dec 24;8(3):271–9. doi: 10.1242/dmm.017889 (PMC4348564; doi:10.1242/dmm.017889)
Supplement: Supplementary Material [file supp_8.3.271_DMM017889.pdf]

Supplementary materials

Figures and Tables

a)

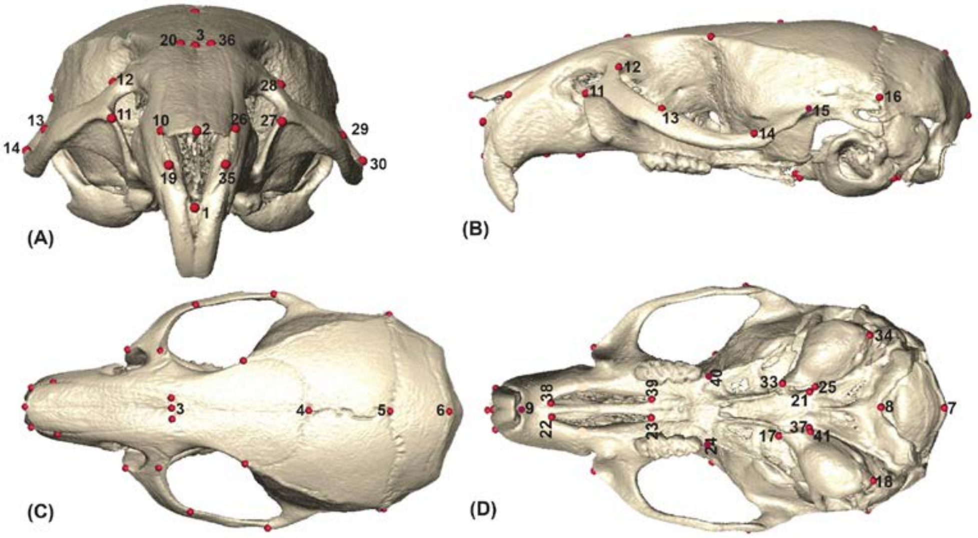

b)

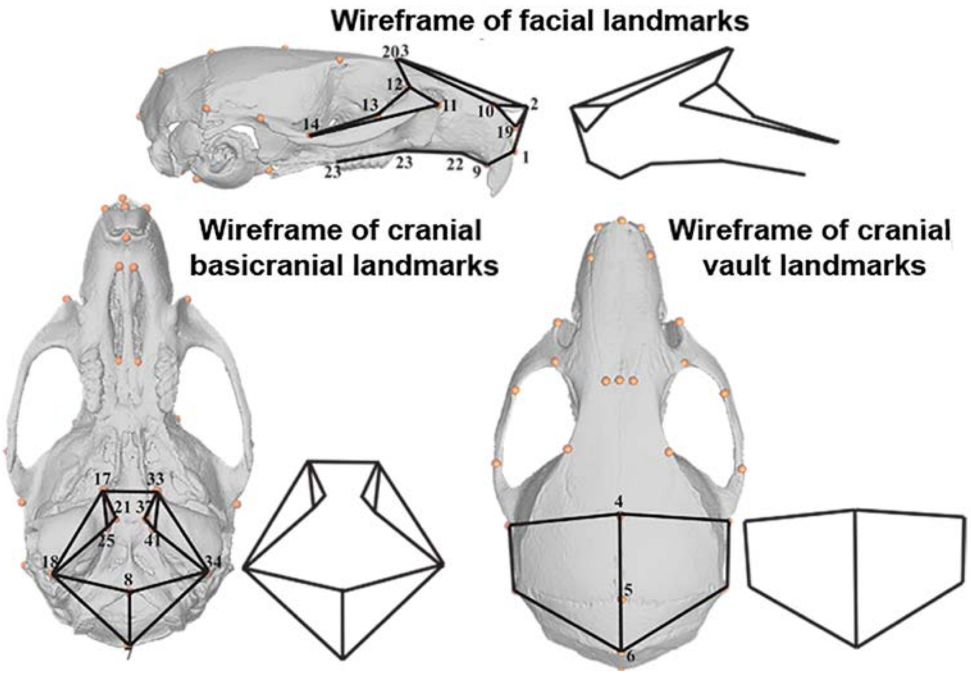

Fig. S1. Landmarks and wireframes used in this study. a) Forty-one landmarks used in the study. (A) Rostral view; (B) Lateral; (C) Dorsal; (D) Ventral. Landmark numbers correspond to definitions in Table S1. Additional views of these landmarks can be accessed at: http://getahead.psu.edu/viewer.html?id=P0_Mouse_Skull
b) Wireframes based on landmark locations used to depict shape changes of the three cranial modules. Views are lateral (facial landmarks), ventral (basicranium) and dorsal (cranial vault).

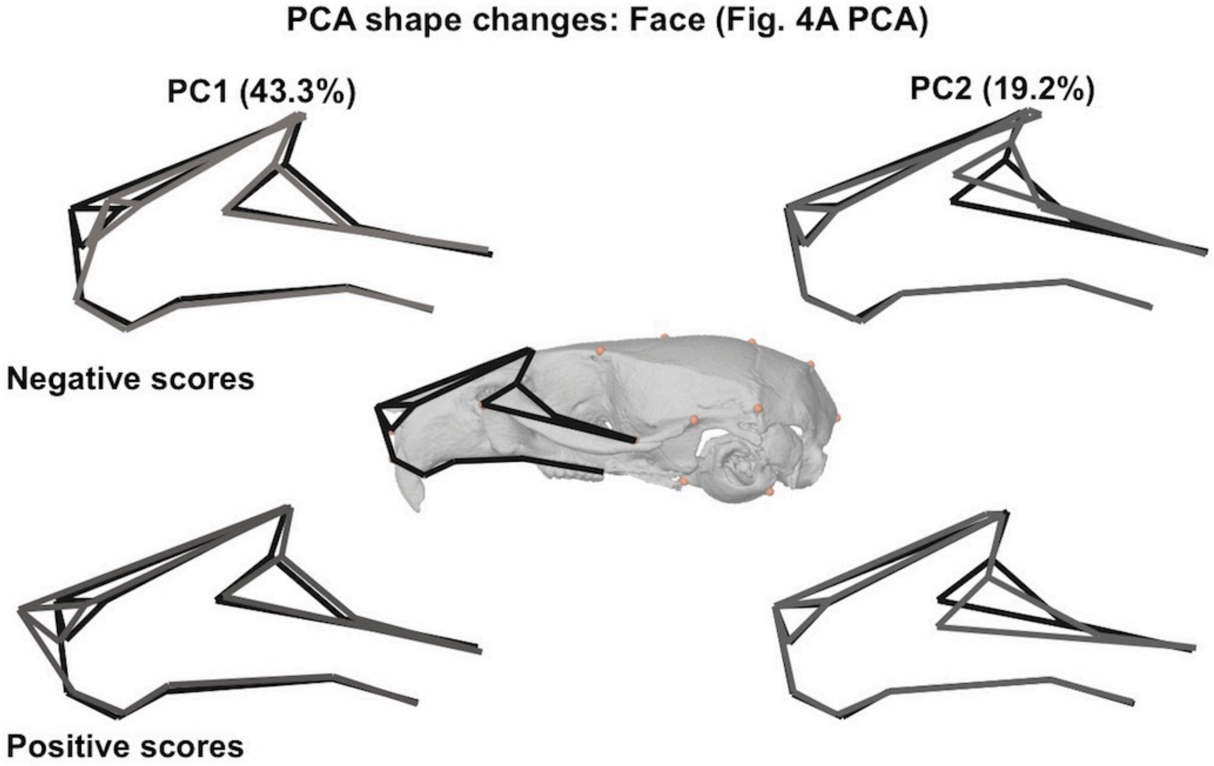

Fig. S2. Wireframes depicting shape changes (in lateral view) along the PC axes shown in Fig. 4A as estimated from the PCA of 24 facial landmarks. The black wireframes represent the overall mean shape of the entire dataset computed from the facial landmarks; Gray wireframes depict the shape changes along the PC axes from the negative to the positive scores. PC1 captures changes mainly in the anterior-posterior dimension, showing a retraction and extension of the snout from the negative to the positive end. PC2 captures changes in the lateral aspects of the facial skeleton, marked by medio-lateral variation in the infraorbital hiatus.

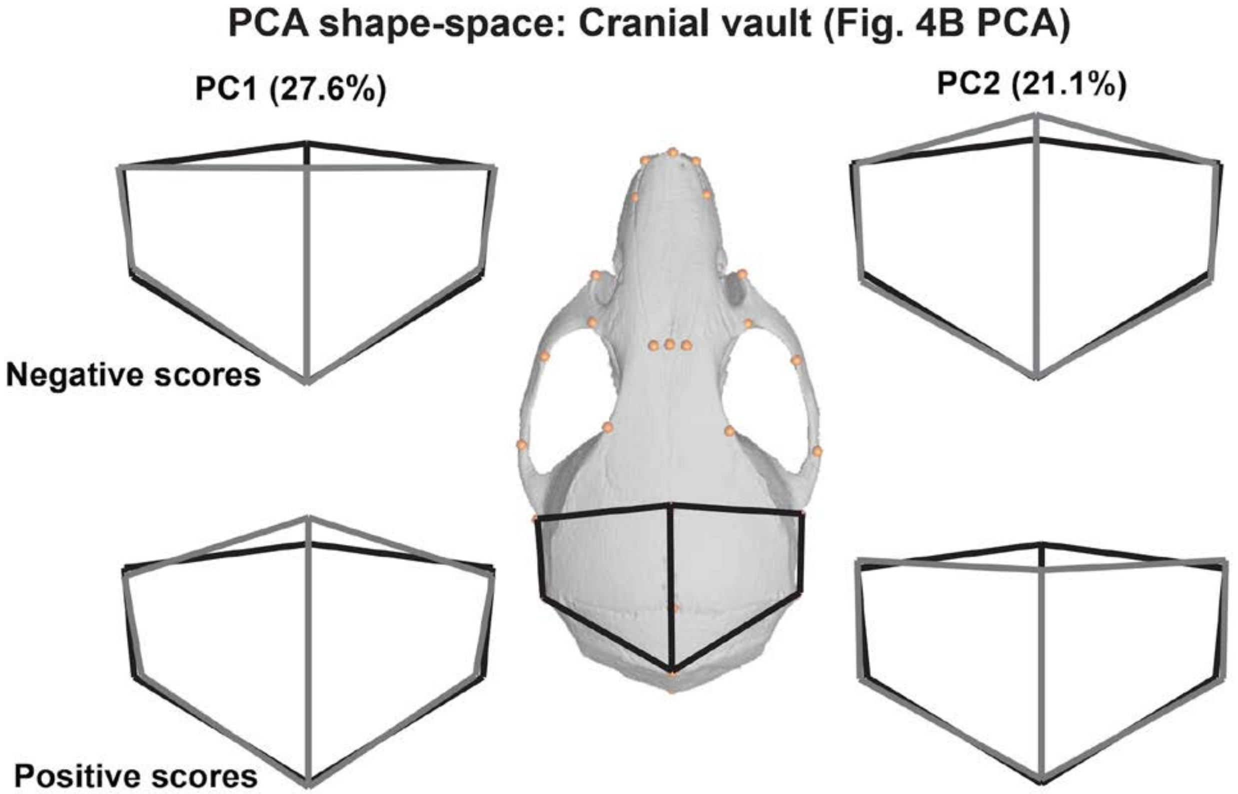

Fig. S3. Wireframes depicting shape changes (dorsal view) along the PC axes shown in Fig. 4B as estimated from the PCA of cranial vault landmarks. Black wireframes represent the overall mean shape of the entire dataset computed from the cranial vault landmarks; Gray wireframes depict the shape changes along the PC axes from the negative to the positive scores. Shape changes along PC1 related to a more globular vs. elongated cranial vault, and PC2 captures changes in the anterior-posterior dimension of the cranial vault.

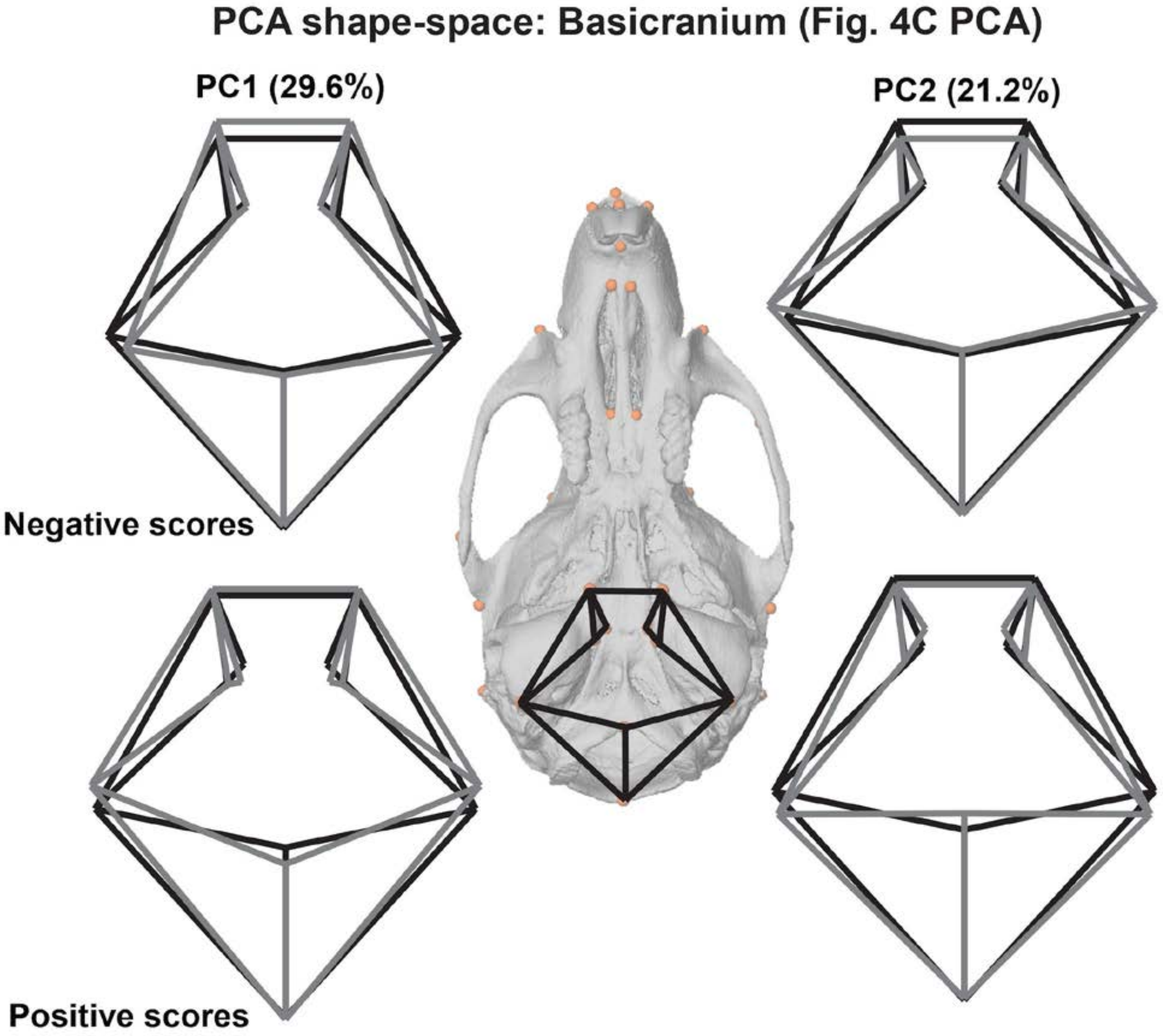

Fig. S4. Wireframes illustrating shape changes of the basicranium (ventral view) along the PC axes shown in Fig. 4C as estimated from the PCA of basicranium landmarks. Black wireframes represent the overall mean shape of the entire dataset computed from the basicranium landmarks; Gray wireframes depict the shape changes from the negative to the positive scores along the respective PC axes. PC1 captures changes in the medio-lateral and anterio-posterior dimension, whereas PC2 relates to changes in the posterior aspect of the basicranium.

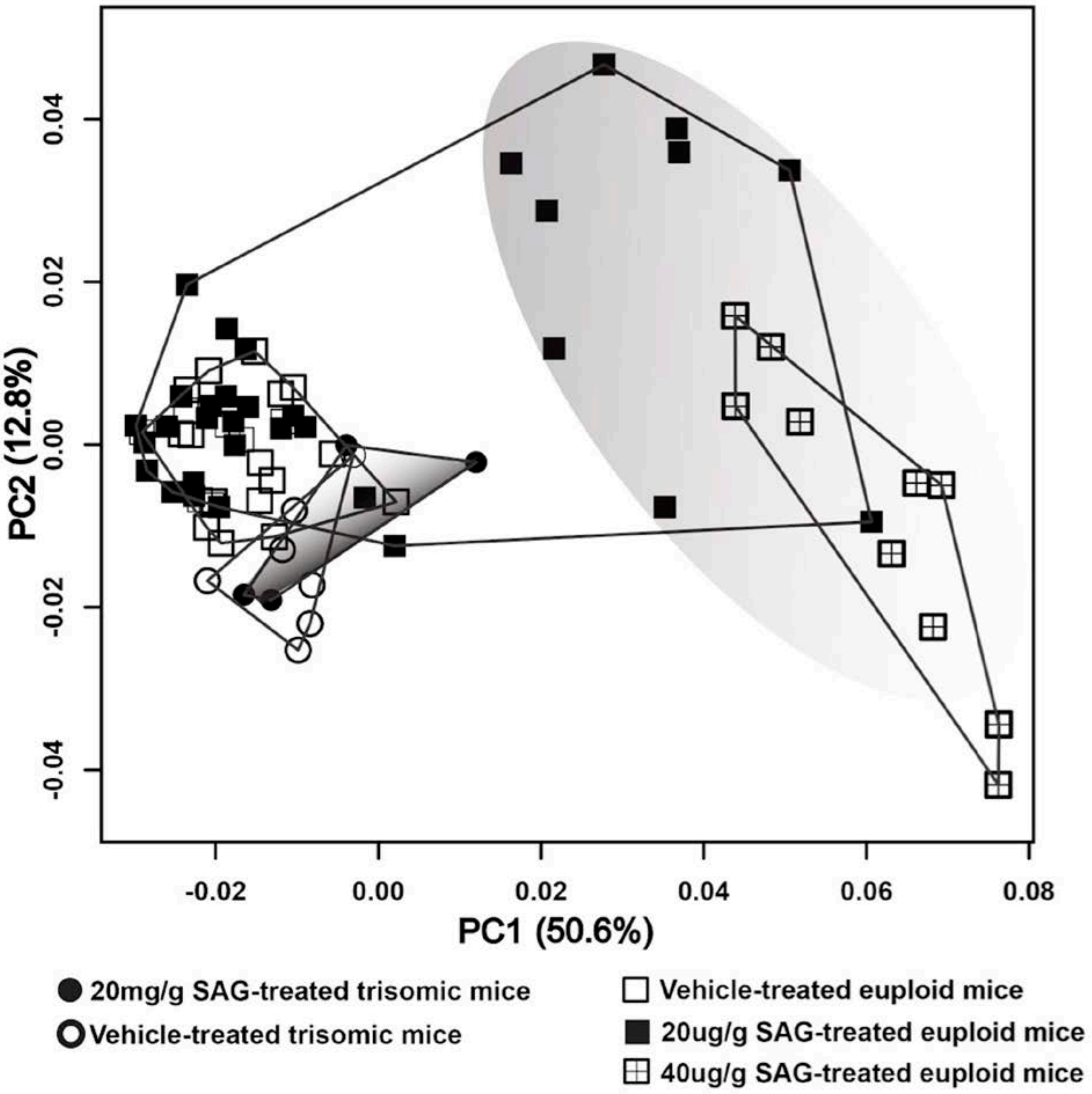

Fig S5. Results of PCA based on Procrustes coordinates of 41 cranial landmarks: Scatter plots of individuals according to their scores along the principle component axes reveal that PC1 separates a subset of 20µg/g SAG-treated and 40 µg/g SAG-treated euploid mice (marked by the gray shaded area) from the remaining SAG-and vehicle-treated euploid animals and from the trisomic groups. The SAG-treated trisomic mice show some variation along PC1, extending towards the affected SAG-treated euploids on the positive end of PC1 (marked by the smaller gray area). Trisomic and euploid mice separate somewhat along PC2 but these differences are masked by the strong morphological affects of the agonist on craniofacial morphology. PC2 mainly captures the within-group variation among the SAG mice, distinguishing between the lower and higher dosed specimens and subtle variation between the trisomic groups and unaffected and vehicle euploid mice.

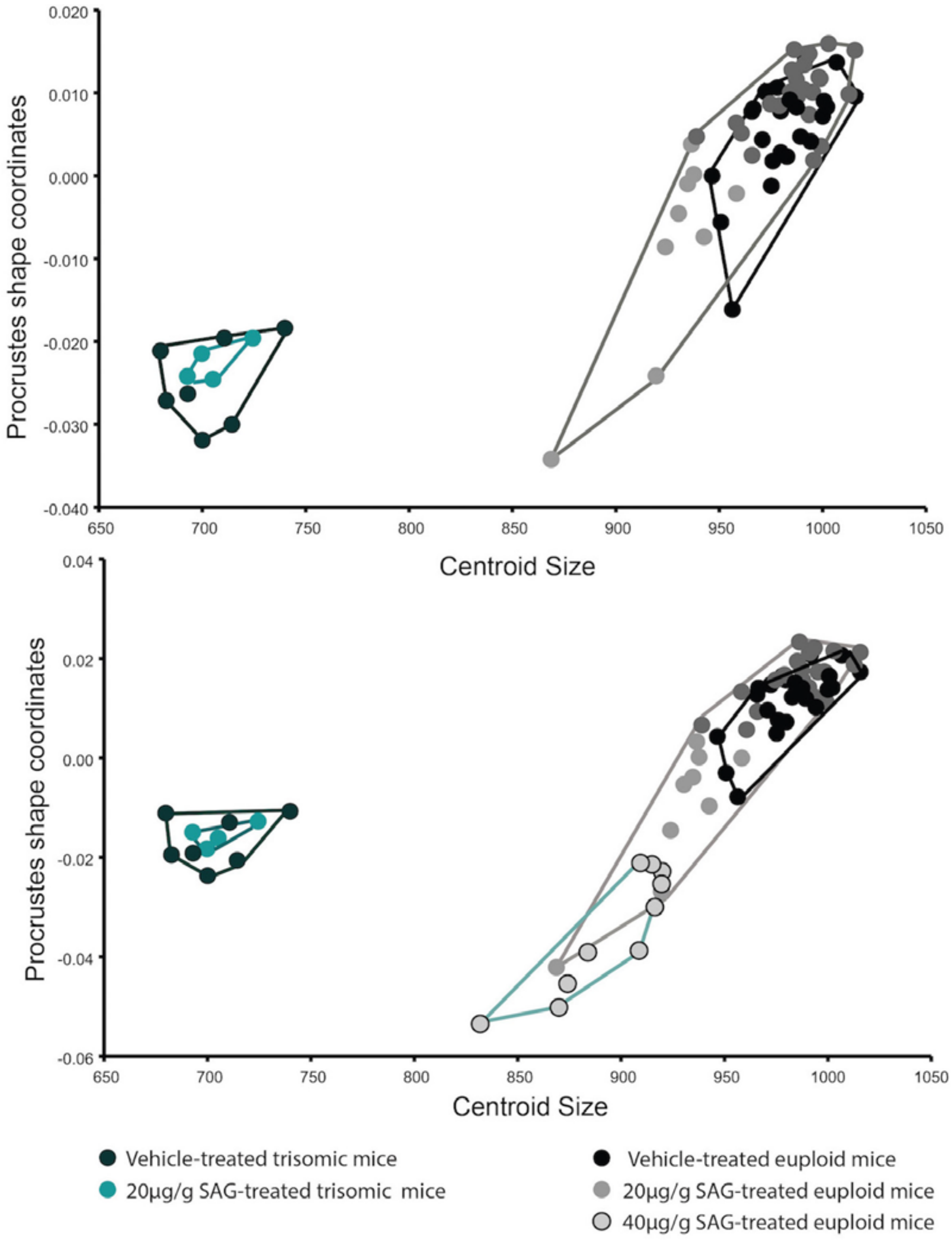

Fig. S6. Multivariate regression analysis of Procrustes shape coordinates on Centroid Size. A) Regression analysis showing clear differences in shape and size between the euploid and trisomic groups. B) Regression analysis that includes the 40µg/g SAG-treated euploid mice confirming a distinct difference between the euploid and trisomic groups but also showing that the 40µg/g SAG-treated mice are distinct in shape and smaller in size than the other euploid mice.

Table S1: Definition of forty-one landmarks used in the study. The landmark numbers correspond to Fig. S1A.

Landmark #	Landmark definition	Cranial region
1	Anterior nasal spine is the most anterior point of interpremaxiallary suture at base of nasal aperture, midline	Face
2	Nasale is the intersection of nasal bones at rostral point, midline;	Face*
3	Nasion is the intersection of nasal bones at caudal point, midline;	Face*
4	Bregma is the intersection of frontal bones and parietal bones at midline;	Vault
5	The intersection of parietal bones with anterior aspect of interparietal bone, midline;	Vault
6	The intersection of interparietal bone with squamous portion of occipital bone, midline;	Vault
7	Opisthion is the midsaggital point on the posterior margin of the foramen magnum, midline;	Base
8	Basion is the midsaggital point on the anterior margin of the foramen magnum, midline;	Base
9	The midline point on the premaxilla between the incisor just anterior of the incisive foramen;	Face
10 & 26	Anterior-most point at intersection of premaxilla and nasal bones;	Face*
11 & 27	Anterior notch on frontal process lateral to infraorbital fissure;	Face*
12 & 28	Intersection of frontal process of maxilla with frontal and lacrimal bones;	Face*
13 & 29	Intersection of zygomatic process of maxilla with zygoma (jugal), superior surface;	Face
14 & 30	Intersection of zygoma with zygomatic process of temporal, superior aspect;	Face
15 & 31	Posterior point at the joining of the squamosal body to zygomatic process of squamosal;	Vault
16 & 32	Intersection of parietal temporal and occipital bones;	Vault
17 & 33	Medial most tip of the tympanic bulla;	Base
18 & 34	Postero-lateral most point on the tympanic bulla;	Base
19 & 35	Antero-superior most tip on the nasal bone;	Face
20 & 36	Point left of nasion intersection of nasal bone and premaxilla;	Face*
21 & 37	Most antero-lateral point on corner of the basioccipital;	Base
22 & 38	Most anterior point of the anterior palatine foramen;	Face
23 & 39	Most posterior point of the anterior palatine foramen;	Face

24 & 40	The posterior-most point on the central anteriorposterior axis of the left molar alveolus;	Face
25 & 41	Most anterior medial point on the left carotid canal.	Base

*Subset of landmarks used in a separate PCA of the face

Table S2: Seventy-six individuals included in the study.

ID	Born	Harvest	Geno	Dam Genotype	Sire Genotype	Sex	Treatment	Euthanized	Image parameters
b5855	5/16/11	8/8/11	B6C3H	JAX B6/C3H	Fertile Male Ts65Dn B6/C3H	M	SAG injection	Anesthetized, Perfusion	16-bit signed; 768 x 768 image size; 0.05mm pixel size; frame 1
b5854	5/16/11	8/8/11	B6C3H	JAX B6/C3H	Fertile Male Ts65Dn B6/C3H	M	SAG injection	Anesthetized, Perfusion	16-bit signed; 768 x 768 image size; 0.05mm pixel size; frame 1
b5851	5/16/11	8/3/11	B6C3H	JAX B6/C3H	Fertile Male Ts65Dn B6/C3H	M	SAG injection	Anesthetized, Perfusion	16-bit signed; 768 x 768 image size; 0.05mm pixel size; frame 1
b5553	4/28/11	7/20/11	B6C3H	Ts65Dn	B6C3H	M	SAG injection	Anesthetized, Perfusion	16-bit signed; 768 x 768 image size; 0.05mm pixel size; frame 1
b6012	6/6/11	8/31/11	B6C3H	JAX B6/C3H	Fertile Male Ts65Dn B6/C3H	M	SAG injection	Anesthetized, Perfusion	16-bit signed; 768 x 768 image size; 0.05mm pixel size; frame 1
b6013	6/6/11	8/31/11	B6C3H	JAX B6/C3H	Fertile Male Ts65Dn B6/C3H	M	SAG injection	Anesthetized, Perfusion	16-bit signed; 768 x 768 image size; 0.05mm pixel size; frame 1
b6015	6/6/11	9/7/11	B6C3H	JAX B6/C3H	Fertile Male Ts65Dn B6/C3H	M	SAG injection	Anesthetized, Perfusion	16-bit signed; 768 x 768 image size; 0.05mm pixel size; frame 1
b6016	6/6/11	9/7/11	B6C3H	JAX B6/C3H	Fertile Male Ts65Dn B6/C3H	M	SAG injection	Anesthetized, Perfusion	16-bit signed; 768 x 768 image size; 0.05mm pixel size; frame 1

b6017	6/6/11	9/7/11	B6C3H	JAX B6/C3H	Fertile Male Ts65Dn B6/C3H	M	SAG injection	Anesthetized, Perfusion	16-bit signed; 768 x 768 image size; 0.05mm pixel size; frame 1
b6019	6/6/11	9/7/11	B6C3H	JAX B6/C3H	Fertile Male Ts65Dn B6/C3H	M	SAG injection	Anesthetized, Perfusion	16-bit signed; 768 x 768 image size; 0.05mm pixel size; frame 1
b6020	6/6/11	8/31/11	B6C3H	JAX B6/C3H	Fertile Male Ts65Dn B6/C3H	M	SAG injection	Anesthetized, Perfusion	16-bit signed; 768 x 768 image size; 0.05mm pixel size; frame 1
b6594	6/26/11	11/7/11	B6C3H	JAX B6/C3H	Fertile Male Ts65Dn B6/C3H	M	SAG injection	Anesthetized, Perfusion	16-bit signed; 768 x 768 image size; 0.05mm pixel size; frame 1
b6595	6/26/11	11/5/11	B6C3H	JAX B6/C3H	Fertile Male Ts65Dn B6/C3H	M	SAG injection	Anesthetized, Perfusion	16-bit signed; 768 x 768 image size; 0.05mm pixel size; frame 1
b6596	6/26/11	11/7/11	B6C3H	JAX B6/C3H	Fertile Male Ts65Dn B6/C3H	M	SAG injection	Anesthetized, Perfusion	16-bit signed; 768 x 768 image size; 0.05mm pixel size; frame 1
b6597	6/26/11	11/7/11	B6C3H	JAX B6/C3H	Fertile Male Ts65Dn B6/C3H	M	SAG injection	Anesthetized, Perfusion	16-bit signed; 768 x 768 image size; 0.05mm pixel size; frame 1
b6598	6/26/11	11/7/11	B6C3H	JAX B6/C3H	Fertile Male Ts65Dn B6/C3H	M	SAG injection	Anesthetized, Perfusion	16-bit signed; 768 x 768 image size; 0.05mm pixel size; frame 1
b5839	5/16/11	8/3/11	B6C3H	JAX B6/C3H	Fertile Male Ts65Dn B6/C3H	M	Vehicle Injection	Anesthetized, Perfusion	16-bit signed; 768 x 768 image size; 0.05mm pixel size; frame 1
b5847	5/16/11	8/2/11	B6C3H	JAX B6/C3H	Fertile Male Ts65Dn B6/C3H	M	Vehicle Injection	Anesthetized, Perfusion	16-bit signed; 768 x 768 image size; 0.05mm pixel size; frame 1

b5849	5/16/11	8/2/11	B6C3H	JAX B6/C3H	Fertile Male Ts65Dn B6/C3H	M	Vehicle Injection	Anesthetized, Perfusion	16-bit signed; 768 x 768 image size; 0.05mm pixel size; frame 1
b5846	5/16/11	8/10/11	B6C3H	JAX B6/C3H	Fertile Male Ts65Dn B6/C3H	M	Vehicle Injection	Anesthetized, Perfusion	16-bit signed; 768 x 768 image size; 0.05mm pixel size; frame 1
b5844	5/16/11	8/10/11	B6C3H	JAX B6/C3H	Fertile Male Ts65Dn B6/C3H	M	Vehicle Injection	Anesthetized, Perfusion	16-bit signed; 768 x 768 image size; 0.05mm pixel size; frame 1
b5840	5/16/11	8/8/11	B6C3H	JAX B6/C3H	Fertile Male Ts65Dn B6/C3H	M	Vehicle Injection	Anesthetized, Perfusion	16-bit signed; 768 x 768 image size; 0.05mm pixel size; frame 1
b5843	5/16/11	8/10/11	B6C3H	JAX B6/C3H	Fertile Male Ts65Dn B6/C3H	M	Vehicle Injection	Anesthetized, Perfusion	16-bit signed; 768 x 768 image size; 0.05mm pixel size; frame 1
b5845	5/16/11	8/10/11	B6C3H	JAX B6/C3H	Fertile Male Ts65Dn B6/C3H	M	Vehicle Injection	Anesthetized, Perfusion	16-bit signed; 768 x 768 image size; 0.05mm pixel size; frame 1
b6036	6/5/11	8/24/11	B6C3H	JAX B6/C3H	Fertile Male Ts65Dn B6/C3H	M	Vehicle Injection	Anesthetized, Perfusion	16-bit signed; 768 x 768 image size; 0.05mm pixel size; frame 1
b6037	6/5/11	8/24/11	B6C3H	JAX B6/C3H	Fertile Male Ts65Dn B6/C3H	M	Vehicle Injection	Anesthetized, Perfusion	16-bit signed; 768 x 768 image size; 0.05mm pixel size; frame 1
b6042	6/5/11	8/24/11	B6C3H	JAX B6/C3H	Fertile Male Ts65Dn B6/C3H	M	Vehicle Injection	Anesthetized, Perfusion	16-bit signed; 768 x 768 image size; 0.05mm pixel size; frame 1
b8755	5/21/12	7/31/12	B6C3H	Ts65Dn	B6C3H	M	SAG injection	Anesthetized Cervical dislocation	16-bit signed; 768 x 768 image size; 0.05mm pixel size; frame 1

b8756	5/21/12	7/31/12	B6C3H	Ts65Dn	B6C3H	M	SAG injection	Anesthetized Cervical dislocation	16-bit signed; 768 x 768 image size; 0.05mm pixel size; frame 1
b8760	5/16/12	7/31/12	B6C3H	Ts65Dn	B6C3H	M	SAG injection	Anesthetized Cervical dislocation	16-bit signed; 768 x 768 image size; 0.05mm pixel size; frame 1
b8761	5/16/12	7/31/12	B6C3H	Ts65Dn	B6C3H	M	SAG injection	Anesthetized Cervical dislocation	16-bit signed; 768 x 768 image size; 0.05mm pixel size; frame 1
b8762	5/16/12	7/31/12	B6C3H	Ts65Dn	B6C3H	M	SAG injection	Anesthetized Cervical dislocation	16-bit signed; 768 x 768 image size; 0.05mm pixel size; frame 1
b8763	5/16/12	7/31/12	B6C3H	Ts65Dn	B6C3H	M	SAG injection	Anesthetized Cervical dislocation	16-bit signed; 768 x 768 image size; 0.05mm pixel size; frame 1
b8764	5/16/12	7/31/12	B6C3H	Ts65Dn	B6C3H	M	SAG injection	Anesthetized Cervical dislocation	16-bit signed; 768 x 768 image size; 0.05mm pixel size; frame 1
b8766	5/16/12	7/31/12	B6C3H	Ts65Dn	B6C3H	M	Vehicle Injection	Anesthetized Cervical dislocation	16-bit signed; 768 x 768 image size; 0.05mm pixel size; frame 1
b8836	6/5/12	8/20/12	B6C3H	Ts65Dn	B6C3H	M	SAG injection	Anesthetized Cervical dislocation	16-bit signed; 768 x 768 image size; 0.05mm pixel size; frame 1
N02	8/6/12	10/16/12	B6C3H	B6C3H	B6C3H	M	SAG injection	Anesthetized Cervical dislocation	16-bit signed; 768 x 768 image size; 0.05mm pixel size; frame 1
N03	8/6/12	10/16/12	B6C3H	B6C3H	B6C3H	M	SAG injection	Anesthetized Cervical dislocation	16-bit signed; 768 x 768 image size; 0.05mm pixel size; frame 1

N04	8/6/12	10/16/12	B6C3H	B6C3H	B6C3H	M	SAG injection	Anesthetized Cervical dislocation	16-bit signed; 768 x 768 image size; 0.05mm pixel size; frame 1
N06	8/6/12	10/16/12	B6C3H	B6C3H	B6C3H	M	SAG injection	Anesthetized Cervical dislocation	16-bit signed; 768 x 768 image size; 0.05mm pixel size; frame 1
N07	8/1/12	10/16/12	B6C3H	B6C3H	B6C3H	M	SAG injection	Anesthetized Cervical dislocation	16-bit signed; 768 x 768 image size; 0.05mm pixel size; frame 1
N09	8/1/12	10/16/12	B6C3H	B6C3H	B6C3H	M	SAG injection	Anesthetized Cervical dislocation	16-bit signed; 768 x 768 image size; 0.05mm pixel size; frame 1
N10	8/1/12	10/16/12	B6C3H	B6C3H	B6C3H	M	SAG injection	Anesthetized Cervical dislocation	16-bit signed; 768 x 768 image size; 0.05mm pixel size; frame 1
N11	8/1/12	10/16/12	B6C3H	B6C3H	B6C3H	M	SAG injection	Anesthetized Cervical dislocation	16-bit signed; 768 x 768 image size; 0.05mm pixel size; frame 1
N12	7/20/12	10/16/12	B6C3H	B6C3H	B6C3H	M	Vehicle Injection	Anesthetized Cervical dislocation	16-bit signed; 768 x 768 image size; 0.05mm pixel size; frame 1
N13	7/20/12	10/16/12	B6C3H	B6C3H	B6C3H	M	Vehicle Injection	Anesthetized Cervical dislocation	16-bit signed; 768 x 768 image size; 0.05mm pixel size; frame 1
N14	7/20/12	10/16/12	B6C3H	B6C3H	B6C3H	M	Vehicle Injection	Anesthetized Cervical dislocation	16-bit signed; 768 x 768 image size; 0.05mm pixel size; frame 1
N15	7/20/12	10/16/12	B6C3H	B6C3H	B6C3H	M	Vehicle Injection	Anesthetized Cervical dislocation	16-bit signed; 768 x 768 image size; 0.05mm pixel size; frame 1

N16	7/20/12	10/16/12	B6C3H	B6C3H	B6C3H	M	Vehicle Injection	Anesthetized Cervical dislocation	16-bit signed; 768 x 768 image size; 0.05mm pixel size; frame 1
N18	7/27/12	10/16/12	B6C3H	B6C3H	B6C3H	M	Vehicle Injection	Anesthetized Cervical dislocation	16-bit signed; 768 x 768 image size; 0.05mm pixel size; frame 1
N19	7/27/12	10/16/12	B6C3H	B6C3H	B6C3H	M	Vehicle Injection	Anesthetized Cervical dislocation	16-bit signed; 768 x 768 image size; 0.05mm pixel size; frame 1
N20	7/27/12	10/16/12	B6C3H	B6C3H	B6C3H	M	Vehicle Injection	Anesthetized Cervical dislocation	16-bit signed; 768 x 768 image size; 0.05mm pixel size; frame 1
N21	7/27/12	10/16/12	B6C3H	B6C3H	B6C3H	M	Vehicle Injection	Anesthetized Cervical dislocation	16-bit signed; 768 x 768 image size; 0.05mm pixel size; frame 1
N22	4/16/13	7/16/13	B6C3H	B6C3H	B6C3H	M	Double SAG injection	Anesthetized Cervical dislocation	16-bit signed; 768 x 768 image size; 0.05mm pixel size; frame 1
N23	4/16/13	7/16/13	B6C3H	B6C3H	B6C3H	M	Double SAG injection	Anesthetized Cervical dislocation	16-bit signed; 768 x 768 image size; 0.05mm pixel size; frame 1
N24	4/16/13	7/16/13	B6C3H	B6C3H	B6C3H	M	Double SAG injection	Anesthetized Cervical dislocation	16-bit signed; 768 x 768 image size; 0.05mm pixel size; frame 1
N25	4/16/13	7/16/13	B6C3H	B6C3H	B6C3H	M	Double SAG injection	Anesthetized Cervical dislocation	16-bit signed; 768 x 768 image size; 0.05mm pixel size; frame 1
N26	4/16/13	7/16/13	B6C3H	B6C3H	B6C3H	M	Double SAG injection	Anesthetized Cervical dislocation	16-bit signed; 768 x 768 image size; 0.05mm pixel size; frame 1

N27	4/16/13	7/16/13	B6C3H	B6C3H	B6C3H	M	Double SAG injection	Anesthetized Cervical dislocation	16-bit signed; 768 x 768 image size; 0.05mm pixel size; frame 1
N28	5/8/13	7/16/13	B6C3H	B6C3H	B6C3H	M	Double SAG injection	Anesthetized Cervical dislocation	16-bit signed; 768 x 768 image size; 0.05mm pixel size; frame 1
N29	5/8/13	7/16/13	B6C3H	B6C3H	B6C3H	M	Double SAG injection	Anesthetized Cervical dislocation	16-bit signed; 768 x 768 image size; 0.05mm pixel size; frame 1
N30	5/8/13	7/16/13	B6C3H	B6C3H	B6C3H	M	Double SAG injection	Anesthetized Cervical dislocation	16-bit signed; 768 x 768 image size; 0.05mm pixel size; frame 1
N31	5/8/13	7/16/13	B6C3H	B6C3H	B6C3H	M	Double SAG injection	Anesthetized Cervical dislocation	16-bit signed; 768 x 768 image size; 0.05mm pixel size; frame 1
N32	4/16/13	7/16/13	B6C3H	B6C3H	B6C3H	M	Vehicle Injection	Anesthetized Cervical dislocation	16-bit signed; 768 x 768 image size; 0.05mm pixel size; frame 1
N33	4/16/13	7/16/13	B6C3H	B6C3H	B6C3H	M	Vehicle Injection	Anesthetized Cervical dislocation	16-bit signed; 768 x 768 image size; 0.05mm pixel size; frame 1
b4356	11/15/10	6/15/11	Ts65Dn	Ts65Dn	B6C3H	M	Vehicle Injection	Anesthetized, Perfusion	16-bit signed; 768 x 768 image size; 0.05mm pixel size; frame 1
b4358	11/15/10	6/15/11	Ts65Dn	Ts65Dn	B6C3H	M	Vehicle Injection	Anesthetized, Perfusion	16-bit signed; 768 x 768 image size; 0.05mm pixel size; frame 1
b5552	4/28/11	7/20/11	Ts65Dn	Ts65Dn	B6C3H	M	SAG injection	Anesthetized, Perfusion	16-bit signed; 768 x 768 image size; 0.05mm pixel size; frame 1

b5837	5/16/11	7/5/11	Ts65Dn	JAX B6/C3H	Fertile Male Ts65Dn B6/C3H	M	Vehicle Injection	Anesthetized, Perfusion	16-bit signed; 768 x 768 image size; 0.05mm pixel size; frame 1
b5838	5/16/11	8/1/11	Ts65Dn	JAX B6/C3H	Fertile Male Ts65Dn B6/C3H	M	Vehicle Injection	Anesthetized, Perfusion	16-bit signed; 768 x 768 image size; 0.05mm pixel size; frame 1
b5848	5/16/11	8/1/11	Ts65Dn	JAX B6/C3H	Fertile Male Ts65Dn B6/C3H	M	Vehicle Injection	Anesthetized, Perfusion	16-bit signed; 768 x 768 image size; 0.05mm pixel size; frame 1
b5850	5/16/11	7/5/11	Ts65Dn	JAX B6/C3H	Fertile Male Ts65Dn B6/C3H	M	SAG injection	Anesthetized, Perfusion	16-bit signed; 768 x 768 image size; 0.05mm pixel size; frame 1
b5852	5/16/11	7/5/11	Ts65Dn	JAX B6/C3H	Fertile Male Ts65Dn B6/C3H	M	SAG injection	Anesthetized, Perfusion	16-bit signed; 768 x 768 image size; 0.05mm pixel size; frame 1
b5853	5/16/11	7/5/11	Ts65Dn	JAX B6/C3H	Fertile Male Ts65Dn B6/C3H	M	SAG injection	Anesthetized, Perfusion	16-bit signed; 768 x 768 image size; 0.05mm pixel size; frame 1
b6647	6/26/11	11/21/11	Ts65Dn	JAX B6/C3H	Fertile Male Ts65Dn B6/C3H	M	Vehicle Injection	Anesthetized, Perfusion	16-bit signed; 768 x 768 image size; 0.05mm pixel size; frame 1
b6656	6/26/11	11/21/11	Ts65Dn	JAX B6/C3H	Fertile Male Ts65Dn B6/C3H	M	Vehicle Injection	Anesthetized, Perfusion	16-bit signed; 768 x 768 image size; 0.05mm pixel size; frame 1
